# Human-Animal Relationship Dysfunction: A Case Study of Animal Hoarding in Italy

**DOI:** 10.3390/ani10091501

**Published:** 2020-08-25

**Authors:** Danila d’Angelo, Francesca Ciani, Alessandra Zaccherini, Simona Tafuri, Luigi Avallone, Serenella d’Ingeo, Angelo Quaranta

**Affiliations:** 1 Department of Veterinary Medicine and Animal Production, University of Naples Federico II, 80137 Naples, Italy; danila.dangelo@unina.it (D.d.); ciani@unina.it (F.C.); stafuri@unina.it (S.T.); avallone@unina.it (L.A.); 2 Instituto Zooprofilattico del Mezzogiorno, Via della Salute, 2, 80055 Portici, Italy; alessandra.zaccherini@izsmportici.it; 3 Department of Veterinary Medicine, Section of Behavioral Sciences and Animal Bioethics, University of Bari “Aldo Moro”, 70121 Bari, Italy; serenella.dingeo@uniba.it

**Keywords:** animal welfare, animal hoarding, obsessive-compulsive disorder (OCD), behavioral and clinic signs, abused dogs

## Abstract

**Simple Summary:**

Animal hording is a psychiatric disorder characterized by the accumulation of animals without providing them with adequate living conditions and the fulfillment of their minimum hygiene and ethological needs. It is now considered as a form of animal cruelty. Here, we present a case of an animal hoarding investigation from Italy that began in 2005 that remains unresolved. We describe the patient’s living and health conditions as well as the legal and animal welfare issues arising from the case. The difficulties that emerged from this case and the involvement of various bodies and agencies highlight the need for a multidisciplinary approach. In fact, this type of problem involves the human, animal, and environmental spheres. Moreover, a holistic approach should be taken and the creation of a National Observatory for Animal Hoarding Disorders could be useful to coordinate stakeholders’ interventions in order to adopt an efficient solution.

**Abstract:**

“Animal hoarding” or “compulsive hoarding of animals” is a psychiatric disease, which has important social implications and a profound influence on animal welfare. To date, this phenomenon has been little investigated and largely unexplored. The present study aims to systematically describe a case of animal hoarding, which remains unresolved. The report refers to a case of a woman suffering from animal hoarding that emerged in 2005. From March 2014 to December 2019, 450 animals were seized over nine different occasions. This disease had significant implications on the welfare of the animals collected, which lived in poor housing and hygiene conditions that frequently led to their death. Since animal hoarding cases involve sanitary, legal, and veterinary aspects, we believe that a multidisciplinary approach is necessary in order to prevent a recurrence and a new accumulation of animals. A holistic approach should be taken according to the One Health principle that involves different stakeholders at every level in order to adopt an efficient solution.

## 1. Introduction

“Animal hoarding” or “compulsive hoarding of animals” is a psychiatric disease that is closely interrelated with animal abuse [1]. To date, this phenomenon has been little investigated and is largely unexplored. The present study, describing a case of recurrent abuse of animals, can make a valuable contribution to a deeper understanding of such a phenomenon. We revealed several aspects involved in the treatment of this disease, which include animal welfare, human psychiatric disease, and social dysfunction issues. Therefore, to identify people at risk of developing this pathology early, a joint intervention of several professionals is required [1]. This could significantly reduce the incidence of the disease and the numerous and consequent cases of the abuse of the animals accumulated.

To our knowledge, this is the first case of animal hoarding that is systematically reported in the scientific field in Italy. The present study describes a complex case of animal hoarding in the Lazio Region (Italy), which first emerged in 2005, following complaints from neighbors, but it remains unresolved. In particular, human living and health conditions are illustrated, as well as legal and animal welfare issues. Moreover, the need for a holistic approach for the treatment of this disease is presented. This includes close collaboration between the different stakeholders involved at every level in order to adopt an efficient solution. The case here reported proves that the animal seizure is fundamental for animal welfare to be safeguarded; however, it is not conclusive. Animal hoarding is a psychiatric disorder with a high recurrence rate and with no defined treatment [2]. A multidisciplinary approach is therefore needed. This should include both the veterinary authorities and the human mental health services in order to address animal welfare issues and for the treatment of obsessive-compulsive disorder (OCD), respectively. The fragmented approach to the case management led to several recurrences, which significantly affected the welfare of the animals involved. The case emerged legally in 2005, but the first legal actions occurred nine years later, in 2014, when animal seizures were ordered by the legal authorities. To date, the case is still unsolved. This study, which describes a unique but emblematic case of animal hoarding in Italy, also aims to raise awareness among the competent authorities in order to draw up a treatment protocol, in the form of guidelines, for the animal hoarding disease. Moreover, a nationally based observatory could collect data from different organizations in order to optimize the surveillance of this pathology and provide greater protection of animal welfare and the psychiatric disease.

### 1.1. Animal Hoarding: Obsessive-Compulsive Disorder (OCD) and Psychiatric Disease

“Animal hoarding” or “compulsive hoarding of animals” is a psychiatric disease, which has important social implications and a profound influence on animal welfare. According to the fourth revised update of the Diagnostic and Statistical Manual (DSM) [3], these characteristics of the hoarding disorder are considered a possible symptom of obsessive-compulsive disorder (OCD) or obsessive-compulsive personality disorder (OCPD). OCD is characterized by intrusive images, thoughts, or obsessions that the patient busily tries to avoid with actions aimed to suppress and to provide relief from the intrusion (compulsions) [4]. Only recently, with the publication of the fifth update of the DSM-5, OCD was removed from that group and a separate nosographic entity was described in a separate chapter, entitled “Hoarding Disorder” (HD), which is included in the new section, entitled “OCD and Related Disorders” [5]. Animal hoarding is considered a special manifestation of HD [6], with a significant impact on the functioning of individuals [5]. Animal hoarding, which is a part of HD, is considered a rather complex phenomenon that causes problems of legal impact and public health [7,8]. The interest of the scientific community on this topic is evident with the increasing number of the case reports published in several countries. For example, in the USA, an increase of 700 to 2000 cases of animal hoarding per year is estimated, but it is still underestimated, since only the most severe cases are considered [2].

### 1.2. Characteristics of Animal Hoarders

The hoarding of animals is characterized by the collection of animals that significantly affects both the standards of personal hygiene and the care of the animals [2]. Animal hoarders are usually unaware of the problems produced by hoarding. Patronek and Nathanson [9] reported that, in some studies, women are disproportionately over-represented in animal hoarding, but the reason for this marked gender disparity is unknown. Since hoarders are typically reclusive and socially isolated, many cases go undetected [10]. The most commonly hoarded animals are dogs. Nathanson et al. [10] observed that the median number of hoarded animals is 39, but more commonly the number exceeds 100 individuals. Consequently, poor living conditions are common. These include the presence of excessive dirt, non-functioning bathrooms, animal wastes, mold, pests, and even animal carcasses [10,11]. The most basic needs of animals, such as food supply and hygiene, are frequently not satisfied and their painful conditions are commonly ignored. Furthermore, an animal hoarder can become defensive if negatively criticized regarding their deficient animal caregiving attitude [12,13]. Animal hoarders are firmly convinced that their actions are harmless and that animals could not survive without their assistance. However, the accumulation of a large number of animals is not, in itself, indicative of being an accumulator [9].

The American Psychiatric Association distinguishes Mental Disorders in Axis I and Axis II disorders: Axis II includes personality (and developmental) disorders, and all others are in Axis I [3].

Broadly speaking, hoarders typically show Axis I and Axis II personality disorders, whereas animal hoarders exhibit only the second [14]. Object and animal hoarders share deviant personalities, difficulties with relationships, social dysfunction, and reclusiveness—indeed they typically live alone. Remarkable differences between the two dysfunctions include the early onset of object hoarding, usually happening in childhood or early adolescence, whereas animal hoarding usually occurs in adulthood [14]. Case reports indicate that between 31% and 100% of individuals hoarding animals also hoard inanimate possessions [15]. Additionally, object and animal hoarders appear to differ in the hygienic conditions of their house. In fact, a minority of object hoarders’ houses have poor hygienic conditions, while animal hoarders’ houses, in almost 100% of cases, present critical hygienic–sanitary conditions, frequently characterized by the presence of feces, urine, and even the carcasses of dead animals [11].

### 1.3. Animal Hoarding: A Social Dysfunction

Animal hoarding is a worldwide phenomenon with a multifactorial nature, which includes social and psycho-pathological issues. These concern mental health problems, animal welfare, and public safety. Therefore, the involvement of mental health and social services, public health and sanitation services, emergency services, animal law enforcement, and veterinary services, which are responsible for the protection of animal welfare, are needed. The costs associated with hoarding cases are often unknown and rather underestimated [9]. Data regarding the efficacy of intervention strategies to reduce this phenomenon are very few [16,17]. The reported recurrence rates are very high, indicating that the current intervention strategies are inefficient [9,18]. The study of this phenomenon with a scientific and methodical approach, which considers the patient follow–up, could be useful for the treatment of this disease. It should also consider prevention, the optimal management of cases, and the health and psycho-social monitoring of the subjects involved. A systematic approach should aim at reducing the costs, but also at safeguarding animal and human welfare, and preventing recurrence. Several cases of animal hoarding that have been described in the USA, Australia, Brazil, Canada, and Spain [18,19,20,21] highlighted the social, legal and bioethical aspects, and considered this psychiatric pathology as an underestimated emerging phenomenon.

## 2. An Italian Case Study

Here, we present the case of a woman living in the Lazio region (Italy), who has suffered from the animal hording disorder since 2005. We will refer to her as Mrs. P for the protection of her privacy as sensitive data.

The case legally emerged after a complaint lodged by the Animal Protection Volunteers Association to the legal authorities. They reported that Mrs. P’s neighbors complained about continuous barking and unpleasant odors coming from Mrs. P’s house. The judicial procedures from 2005 until December 2019 are reported below, together with the clinical and behavioral aspects of the animals hoarded. In the judicial procedure, the Animal Protection Volunteers Association brought civil action.

### 2.1. Judicial Procedure

Since November 2005, Mrs. P has been accused of several crimes: the incompatible possession of animals, for having detained numerous dogs in conditions that are incompatible with their nature; animal abuse (Article 544-ter of the Italian Criminal Code); the abandonment of animals (Article 727 of the Italian Criminal Code)—in Italy, the abandonment of animals is related to people who abandon animals but also who keep animals in living conditions that are incompatible with their nature; violation of seals (Article 349 of the Italian Criminal Code); failure to comply with the order to move the animals from her house (Article 650 of the Italian Criminal Code); threats addressed to a public official (veterinarian) (Article 336 Italian of the Criminal Code). Some of the judicial proceedings were sentenced, others are close to the definition of the first degree, therefore, to date, the judicial proceedings are not concluded. All the precautionary measures issued were validated by the investigating judge and confirmed by the Review Court.

Mrs. P was definitively sentenced twice (in 2008 and 2018) for the crimes referred to animal abuse, in addition to other related crimes (articles 349, 646, and 648 of Italian Criminal Code).

Over the years, several ordinances have been issued by the Mayor of the Municipality (the highest health authority, required to take custody of animals seized in its territory), which prohibited the possession of animals, even temporarily, in Mrs. P’s house but also in the entire municipal area. She was also ordered to destroy illegal facilities that she built to host the animals (i.e., cages and external structures).

All the seizures, with the exception of those made in 2014, were carried out following the complaints filed by the Animal Protection Volunteers Association, which was also entitled to take care of some of the seized animals.

Mrs. P accumulated over 450 animals (especially dogs, but also cats and horses), raising significant public hygiene issues, since several animals suffer from zoonotic infectious diseases. Despite all the seizure orders and the ongoing proceedings, Mrs. P continued to illegally detain animals, obtaining new ones through the reproduction of animals hosted in her house, the adoption of stray animals, and through online adoptions (through social media from owners who wanted to give their pet away).

### 2.2. Legal Figures and Stakeholders

The authorities involved in the management of an animal hoarder case are mainly the following: the mayor or his delegate; the guarantor for animal rights and Animal Rights Office, if they are present in the municipality; the local police; the services provided by the ASL (Local Sanitary Assistance), belonging to the National Health System; veterinary services; social services; hygiene and public health services; the services and mental health centers managed by the ASL; zoophilic guards; animal welfare associations; mental health associations; the Carabinieri; the Fire Brigade.

The intervention of the national and local veterinary services is crucial for the management and treatment of the animal hoarding problem. In particular, veterinary services play an important role in suspecting, detecting, and identifying people with such a relationship disorder, but they are also in charge of actively monitoring specific local territories and managing animal welfare issues in such areas. Reinisch [20] indicates that specific guidelines can help veterinarians to identify a client as a potential hoarder. Among the factors that could anticipate the development of the animal hoarding disease, the most common are the owner’s lack of interest in animal care, no regular vaccinations, or the presence of untreated parasitosis in the animals. The holding of a high number of animals is not the only symptom that must be considered, since there is not always a direct correlation between the number of animals held and to be an animal hoarder.

The establishment of a network between freelancers and institutional veterinarians for the sharing of data about people potentially suffering from this disorder and the animals involved could constitute an efficient means for the identification and the management of these patients. Their cooperation with all the other stakeholders, which could be coordinated by a national observatory, could be necessary for the effective monitoring and rapid sharing of data. Among the various detected behaviors shown by owners that could potentially develop the animal hoarding disorder, there is also the frequent change in the referent veterinarian for basic health needs in order to avoid suspicion [14].

In most cases, the approach to the problem is fragmented, leading to an ineffective intervention, with both high costs and high recurrence rates. In other words, the current approach is not effective in preventing the recurrence of the problem and for reducing the serious threat for both animal and human health.

### 2.3. Costs

An important element is represented by the considerable costs estimated in an “Animal Hoarding” case, which include: the recovery and transfer of animals; removal of waste; pest control; demolition of illegal structures/restoration of places; animal custody and assistance; medical/veterinary expenses; intervention costs for offices, agencies, services, and bodies involved; court costs [9].

What should be clear is that animal hoarding impacts entire communities and, regardless of the size of the case, requires effort from many different departments for an effective resolution. Animal sheltering organizations are clear primary players that are directly impacted by animal hoarding.

In Italy, the costs of dog maintenance in shelters are supported by the municipality. The Regional Center of Veterinary Urban Hygiene (CRIUV) of the Campania Region has established that the maintenance of each animal in shelter amounts to approximately 1500–1800 €/year/dog, plus the costs of any clinical and behavioral interventions, aimed at improving the adoptability of abused dogs.

## 3. Results

### 3.1. Clinical and Behavioral Problems in the Animals Seized

The information regarding the number of animals and the percentages of clinical and behavioral problems described here come from the reports drafted by the veterinarian in charge for each seizure.

All the seized animals accumulated by Mrs. P. showed similar clinical and behavioral problems. One of the most commonly observed characteristics was marked muscle atrophy, especially of the hind limbs, with a severe difficulty in walking. This clinical symptom could be related to prolonged inactivity/immobilization due to overcrowding, which caused the animals to lie in a position of forced decubitus. The alopecic areas observed were in the same locations in all the animals seized and were compatible with the hair loss and the formation of a skin callus, due to constant contact with a hard surface (i.e., pet carrier and iron boxes).

All the subjects were in a state of severe malnutrition and dehydration and some of them were cachectic (a sign of physical deterioration and prolonged malnutrition over time) and suffered from endo- and ecto-parasitosis.

Despite the fact that the animals lived with humans, they were wary of human manipulation and fearful towards sudden noises or gestures. They also showed signs of discomfort and insecurity towards humans. Moreover, the animals showed clinical signs of malnutrition and several injuries that could have developed over a considerable and prolonged amount of time, suggesting that their living conditions seriously affected their mental and physical health.

Marked coprophagy behavior was also observed. This could be related to prolonged starvation, becoming a habit first developed over time and then stereotyped.

### 3.2. Seizure Data

**Figure 1:**
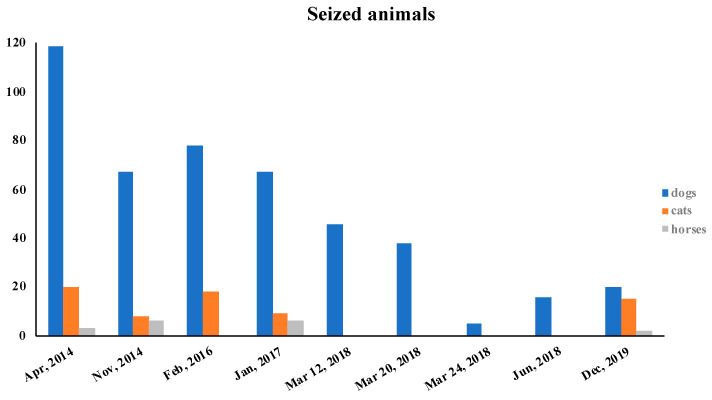
The graph represents the number of animals, divided according to species, subject to the various seizures carried out from April to December 2019.

The competent authorities carried out nine seizures of the held animals, from March 2014 to December 2019, as shown in Figure 1. The seized animals included not only dogs, but also domestic cats and horses.

**Figure 2:**
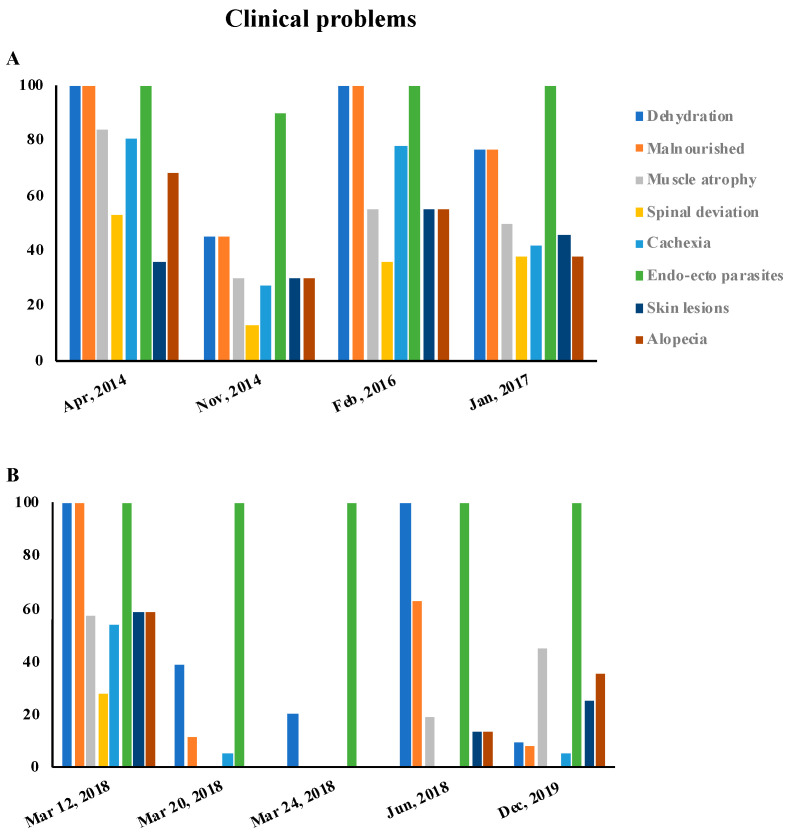
The graphs show the percentages of clinical problems encountered in dogs subject to seizures. (**A**): the data refer to the seizures from April 2014 to January 2017; (**B**): the data refer to the seizures carried out from March 2018 to December 2019.

**Figure 3:**
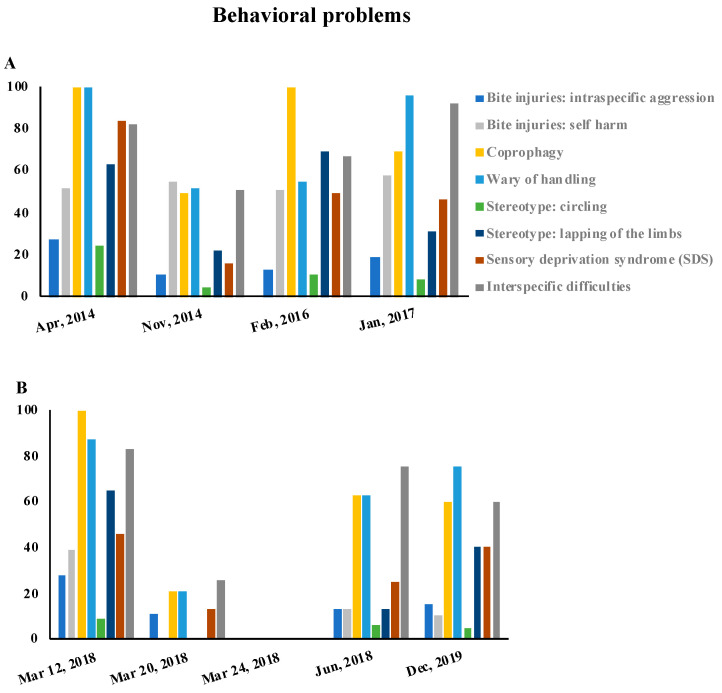
The graphs show the percentages of behavioral problems found in dogs subject to seizures. (**A**): the data refer to the seizures from April 2014 to January 2017; (**B**): the data refer to the seizures carried out from March 2018 to December 2019.

The clinical and behavioral problems observed after the seizures carried out from March 2014 to December 2019 are reported, as percentages, in Figure 2 and Figure 3, respectively.

The presence of the clinical and behavioral problems was closely related to the length of the period of the animals’ detention. In March 2018, three seizures took place. The animals were therefore held for a very short period of time. As a consequence, we registered a low percentage of clinical and behavioral problems in those animals, as shown in Figure 2B and Figure 3B. An extremely serious finding was that Mrs. P was able to obtain new animals in a short period of time through various channels, including social networks.

In all the seizures, dehydration and malnutrition were the most common clinical signs, with high percentages (Figure 2, Figures S1, S3, S6 and S7). Moreover, the presence of endo- and ectoparasites, diagnosed by a veterinarian, was constant in all the seizures for 100% of the animals. Muscle atrophy (Figure 2, Figures S1, S3, and S7) and cachexia (Figure 2, Figures S3, S6, and S7) were generally present with a low percentage and mainly observed in dogs forced into cages without the possibility of free movement.

Skin lesions and alopecia (Figure 2, Figures S2–S7) were found in a high percentage in several seizures. They were related both to the presence of ectoparasites and, most importantly, to prolonged detention in cages. The younger animals also showed spinal deviation in a higher percentage (Figure 2, Figures S2–S5).

Among the behavioral problems, the coprophagia was the most common sign observed (Figure 3). As described above, it was associated with both the clinical situation of malnutrition and with the impossibility of elimination outside of the cages.

Bite injuries related to self-injuries and intraspecific aggression (Figure S8) have also been found. However, these lesions might have different origins and causes (Figure 3). Self-injuries were mainly located in the front limbs and were produced by an intentional act of tissue destruction. Bite injuries, instead, were caused by intraspecific aggressions (Figure S8) due to a severe deficit in the socialization process.

Stereotypes, such as circling and lapping of the limbs, were diagnosed in almost all the seizures. Behavioral signs were caused by the subjects’ restricted living conditions, which also affected the animals’ abilities to cope with stress and discomfort (Figure 3).

In the dogs that were held for longer periods, a wariness of human handling was observed (Figure 3). This could have been related to a lack of interspecific social interactions for long periods, which also includes handling by the owner.

Sensory deprivation syndrome (SDS) (Figure 3) was also observed in adult dogs who had lived in a hypo-stimulating environment, and resulted in a deficit of their abilities to manage new situations.

## 4. Discussion and Critical Points in the Case Management

Here, we described the first case study of animal hoarding in Italy with the purpose of a descriptive and methodological approach. We described the typical features of animal hoarders, their modus operandi, and the social, judicial, health, and veterinary medical aspects involved in such a psychiatric disorder.

The case emerged in 2005 but remains unresolved. Several legal and medical interventions have been carried out in the last few years. However, they were not effective. Despite the numerous recommended medical therapies and the different legal actions (i.e., seizures), to date, the problems related to the accumulated animals’ health and welfare, as well as the treatment of Mrs. P’s disorder still need to be addressed. The accumulation of animals produced clear consequences on their physical and mental health that should be considered when approaching an animal hoarding problem and included in the treatment plan.

### 4.1. Psychological Abuse of Seized Animals

Many of the seized animals presented skin lesions caused both by self-directed licking activity and other subjects’ direct aggressions. The self-injuries were mainly located on the front limbs and were related to self-redirected activity motivated by the high level of stress experienced by the accumulated animals. The self-injuries may function as a coping strategy and can be considered as an attempt to increase the release of endorphins, as reported in human medicine [22]. Bite-related injuries, instead, were caused by intraspecific aggressions (Figure S8) that were related to the deficit in the socialization and the condition of environmental and social deprivation in which the animals lived. This resulted in the lack of the development of coping strategies for facing emotional situations, especially for the management of fear. McMillan et al. [23] also observed high levels of fear in abused dogs, which exhibited fearful-related behavior toward strangers (unfamiliar people) and towards other dogs.

As previously described by Pageat [24], in SDS (sensory deprivation syndrome) one of the main elements is the deficient management of sensory information present in the environment: the behavioral development of the puppy in a hypostimulating environment generates behavioral responses marked by fear. The role played by the mother is fundamental in order to generate an adequate level of sensory homeostasis and make the homeostatic system functional. The puppy will explore its surroundings and learn how to respond to a high number of stimuli only when the attachment bond and the emotional filter are functional. The mother–puppy attachment relationship aids the puppy to form an adequate emotional profile, a social relational model in the acquisition of self-control, and a behavioral style capable of inserting species-specific communication modules. When the style of attachment is impaired, all these factors could give rise to dysfunctional cognitive behavioral profiles. An interesting study has shown that the owner is the reference and care figure of the animal. Siniscalchi et al. [25] showed that, in controlled experimental conditions, the dog–owner relationship is based on a complex and well-established relationship that can be defined as “attachment”.

It is interesting to note that several seized dogs demonstrated a fear-related behavior profile: they were wary of human handling and showed intra- and interspecific aggressive behavior. An increased level of fear of unfamiliar humans would be an expected effect of certain forms of abuse, especially when there is a direct and negative interaction between a human and the abuse [23]. Fear generalization is a well-described consequence of the psychological trauma in animals in an experimental setting [26,27]. In the present study, young animals showed dysfunctional traits related to environmental hypo-stimulation, and the higher percentages of behavioral disturbances are related to the time of detention and, therefore, to the abuse of the animal (Figure 3). Trocmé and colleagues [28] showed that abused dogs exhibited high levels of aggression toward both unfamiliar people and other dogs. In young animals, stressful experiences can produce behavior disorders that are similar to those seen in human abuse victims, including aggression [29,30].

Further studies have shown that the owners’ personality traits, which include a high level of neurosis, lack of openness, and conscientiousness, are related to the style of the owners’ interactions with their dogs and their practices of management. Behavioral problems, such as aggression, anxiety, and excitability [31], have been shown to be associated with the use of positive punishment or advised methods of behavioral control. In such situations, the clinical and behavioral signs of stress increase, whereas the ability to learn and willingness to interact with strangers decrease [32]. The relationship between owner personality and psychological status and the behavior of companion dogs is mediated by the quality or style of the owner’s interactions with the dog. It has been found that people suffering from OCPD, who perceive reality in an altered way [12], generate a strong dysfunctional relationship with their dogs.

### 4.2. Critical Points in the Case Management

We registered a lack of collaboration between the different involved stakeholders for the management of the analyzed case. The main problems observed were the lack of agreements in the plan for the interventions and different approaches to the problem, as well as the lack of organization in the authorities’ interventions (i.e., who should act and how) [33].

Despite the numerous legal actions adopted since 2005, Mrs. P repeated the crime and the judicial authority seized more than 450 animals. The lack of a clear and decisive intervention of the local health services and the judicial police led Mrs. P to accumulate new animals in her house and external structures she managed. This had a great impact on the welfare of the hosted animals.

The hoarder characteristics here reported are in line with the typical animal hoarder features previously described in the literature: the age of the problem onset, the loneliness of the hoarder, the poor hygienic conditions in which the patient and its animals lived, were all features found in this case. Among the legal intervention, it is interesting to note that the house of Mrs. P was confiscated. The reasons that led to this measure were related to the poor hygienic conditions found there. Many dogs were closed in the dark in bathrooms or tied to radiators in the house with no possibility of movement, or they were tied up in cages by their waists. Mrs. P and her animals’ living conditions might have worsened over the years. As Patronek and Nathanson [9] reported, the hoarders might view their animals as extensions of themselves, not really as separate beings. This situation makes the person unable to empathize with the animals or understand that they have their own needs. In other words, hoarders usually neglect themselves and, since they consider the animals as projections of themselves, they cannot perceive the poor health and ethological conditions in which they live. Furthermore, it is possible that animal hoarders practice the dissociation that leads the patient to live in a “parallel” but not real world. This causes the lack of awareness of their degrading living conditions [12]. When a person with a negative affinity for animals actively searches for contact with animals, deliberate abuse is likely to occur. However, the accumulation of animals occurs in the presence of a strong positive bond between the hoarders and their animals. Nevertheless, this relationship is dysfunctional and generally results in the satisfaction of human needs but not those of the animals [1]. Although the seizures of animals are statutory, they are generally ineffective and do not constitute an efficient deterrent for further accumulation. On the contrary, it produces the further psychological suffering of the hoarders, causing them to actively search for other animals in order to relieve those unpleasant feelings.

The failure of the described case is also due to several delays of the judicial system. It is known, in fact, that Italian legal procedures are very long and usually several years are needed for final judgment. Our aim was to give emphasis to the emerging problem of animal hoarding in Italy so that the legislative system can promulgate ad hoc laws in order to speed up the procedures, to reach conclusions in a shorter time, and to avoid patients’ recidivisms, minimizing the suffering of abused animals.

We strongly believe that the establishment of a National Observatory for the Animal Hoarding Disorders is necessary to coordinate stakeholders’ interventions in a standard and homogeneous action plan. Its mission would be the following:

- To collect, promote, exchange, and process data;
- To create guidelines for the stakeholders involved;
- To inform and sensitize public opinion through different networks;
- To organize training and update courses for national health and veterinary services;
- To facilitate judicial proceedings, which should aim at protecting the animals involved.

In the holistic view of One Health, this approach could be a winning strategy. A similar institution has been already established in the US (i.e., the Hoarding of Animals Research Consortium (HARC) [34]) aimed at defining and better understanding the problem of animal hoarding.

## 5. Conclusions

The accumulation of animals is a complex phenomenon that involves different subjects—animals and hoarders, as well as the entire society. As a consequence, several offices and stakeholders are involved and are in charge of identifying an efficient strategic plan for solving this problem. Since these cases have legal consequences and include several aspects of public health, safety, and environmental protection, the involvement of different professionals and skills is required.

The lack of a convergence and common framework often results in the patient’s recurrence and can cause the severe impairment of the accumulated animals’ welfare. Given the compulsive nature of animal hoarders and the extreme reluctance to accept help, rapid and long-lasting changes in their behavior do not frequently occur. Data collection and banking, coming from officials and local and national institutions is therefore fundamental for managing the information related to each case of animal hoarding, at both a regional and national level. The information exchange would be another crucial factor for case management in order to develop an efficient strategy to solve the problem and for significantly reducing the recurrence rate. Moreover, it would be desirable that the legislative system be able to simplify the legal process through ad hoc laws. Finally, the establishment of a National Observatory for the Animal Hoarding Disorders could be, therefore, a useful means to coordinate the stakeholders’ interventions for a standard and homogeneous action plan.

## Supplementary Materials

The following are available online at https://www.mdpi.com/2076-2615/10/9/1501/s1, Figure S1: Seized dog in April 2014, Figure S2: Seized dog in April 2014, Figure S3: Seized dog in April 2014, Figure S4: Seized dog in February 2016, Figure S5: Seized dog in February 2016, Figure S6: Two seized dogs on 12 March 2018, Figure S7: Seized dog on 12 March 2018, Figure S8: Seized dog on 12 March 2018.
