# Supplementary material for: Human-Animal Relationship Dysfunction: A Case Study of Animal Hoarding in Italy
Source: Animals (Basel). 2020 Aug 25;10(9):1501. doi: 10.3390/ani10091501 (PMC7552271; doi:10.3390/ani10091501)
Supplement: Supplementary file 1 [file animals-10-01501-s001.pdf]

Supplementary Information

Title of Manuscript:

Human-animal relationship dysfunction: a case study of animal hoarding in Italy

Authors:

Danila d'Angelo[1#], Francesca Ciani[1#], Alessandra Zaccarini[2], Simona Tafuri[1], Luigi Avallone[1], Serenella d'Ingeo[3], Angelo Quaranta[3]

[1]Department of Veterinary Medicine and Animal Production, University of Naples Federico II, 80137 Naples, Italy; danila.dangelo@unina.it (D.d.); ciani@unina.it (F.C.); stafuri@unina.it (S.T.); avallone@unina.it (L.A.)

[2]Instituto Zooprofilattico del Mezzogiorno, Via della Salute, 2 80055 Portici, Naples, Italy; alessandrazaccherini@gmail.com (A.Z.)

[3]Department of Veterinary Medicine, Section of Animal Physiology and Behaviour, University of Bari "Aldo Moro", 70121 Bari, Italy; serenella.dingeo@uniba.it (S.d.); angelo.quaranta@uniba.it (A.Q.).

[#]The authors contributed equally to this work.

* Correspondence: angelo.quaranta@uniba.it; Tel.: +39-080-544-3927

Supplementary Figures

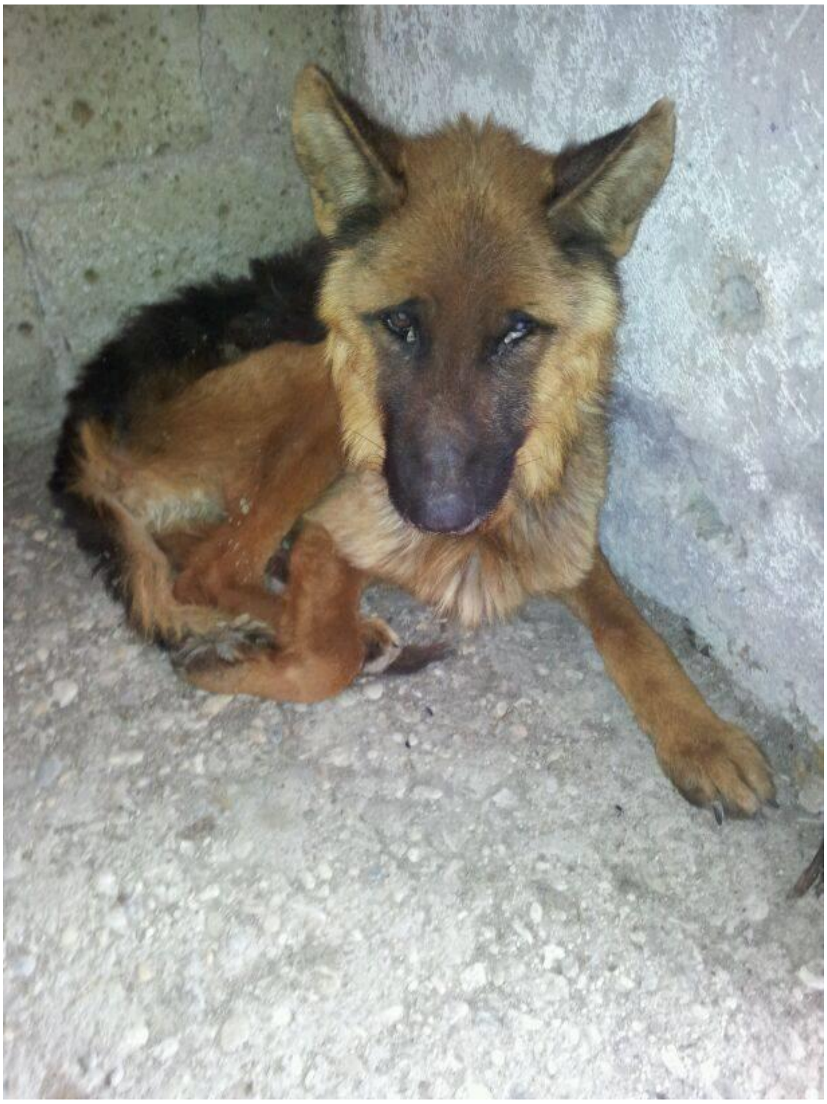

Figure S1. The photo shows a seized dog in April 2014. The state of severe cachexia due to prolonged malnutrition and dehydration is highlighted. The posterior train has muscle hypotrophy.

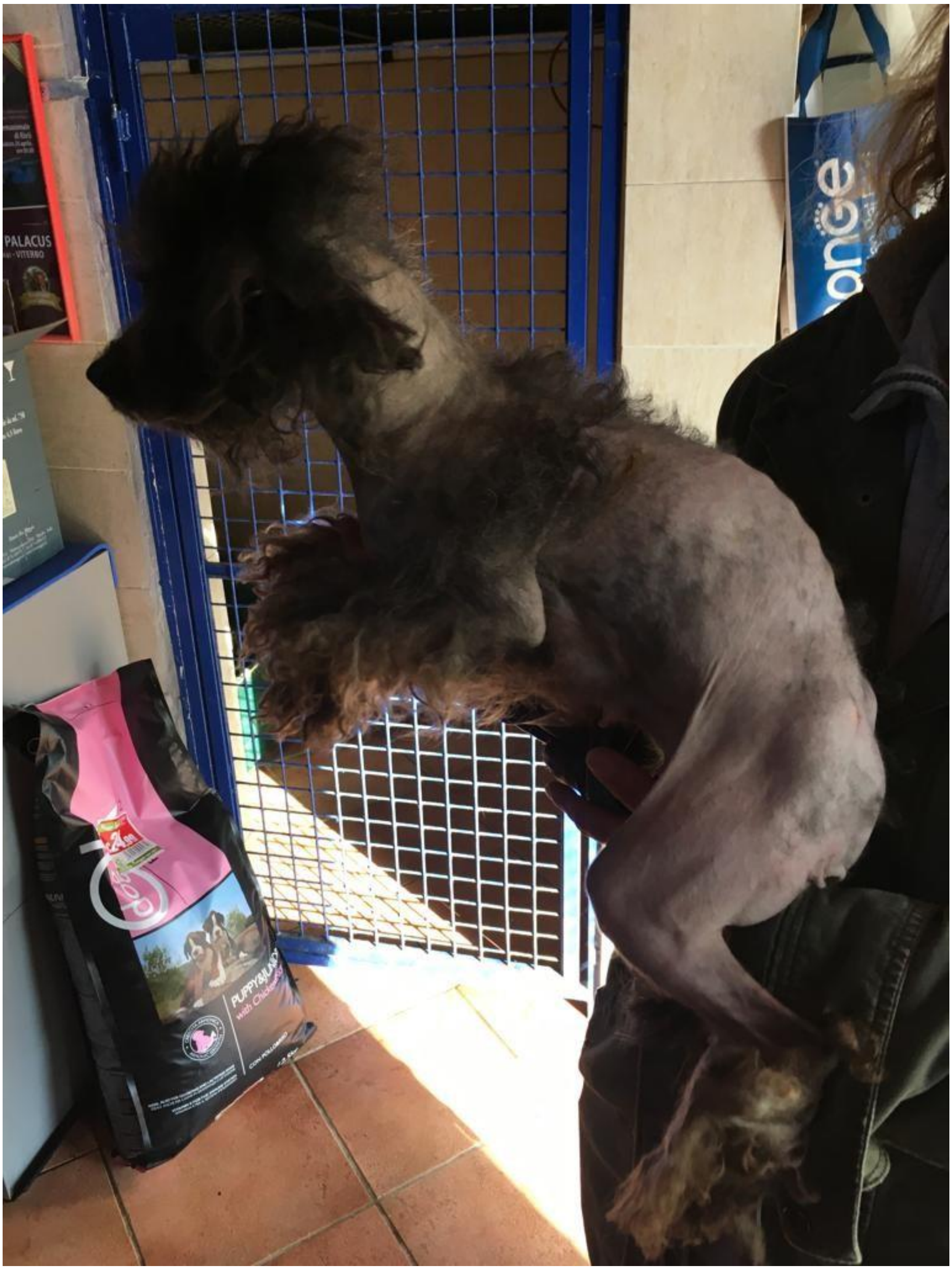

Figure S2. The dog in the photo represents another subject seized in April 2014. The animal shows spinal deviation, due to the prolonged detention in small cages, marked and widespread alopecia.

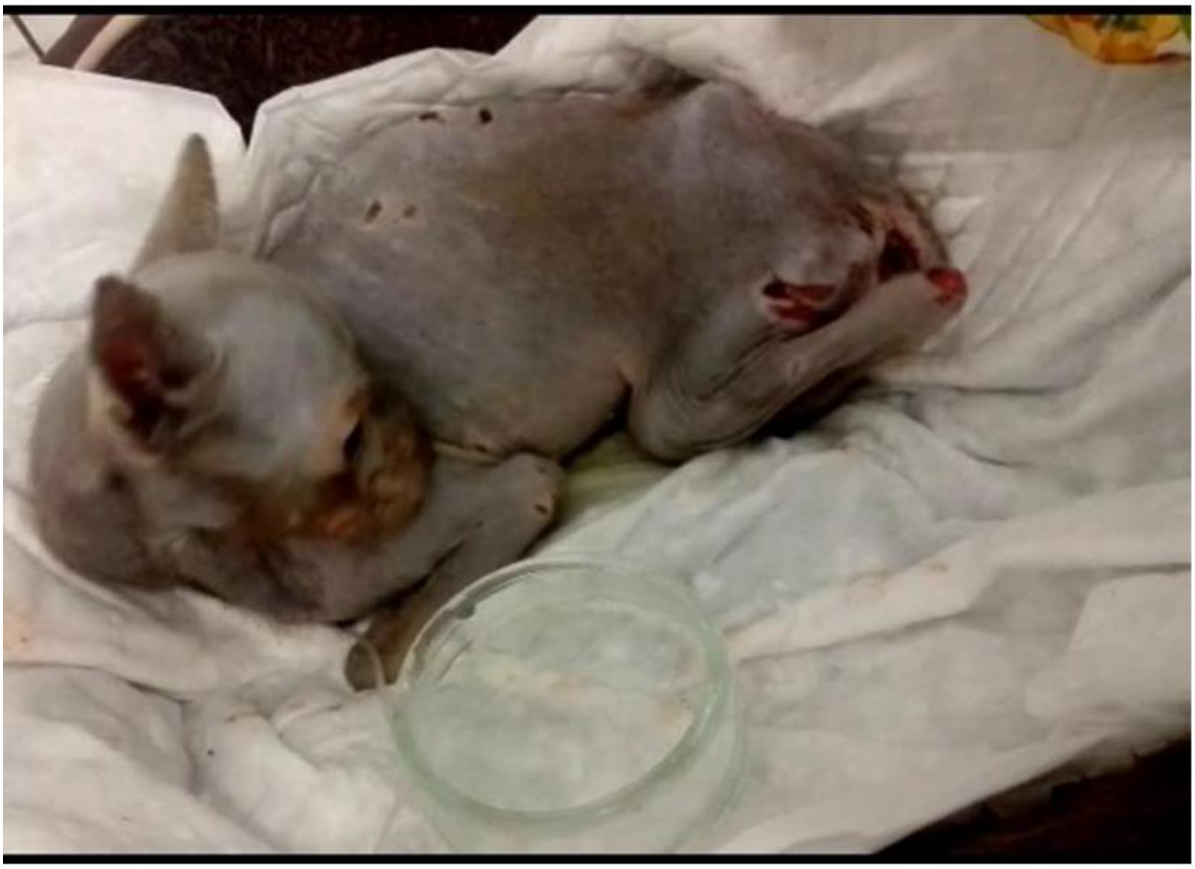

Figure S3. The dog in the photo subject to the seizure that took place in April 2014 shows serious impairment of the general state, a pronounced deviation of the spine, a marked and widespread alopecia, a serious state of malnutrition and dehydration that led the animal to a marked cachectic state. Marked muscle hypotrophy is evident.

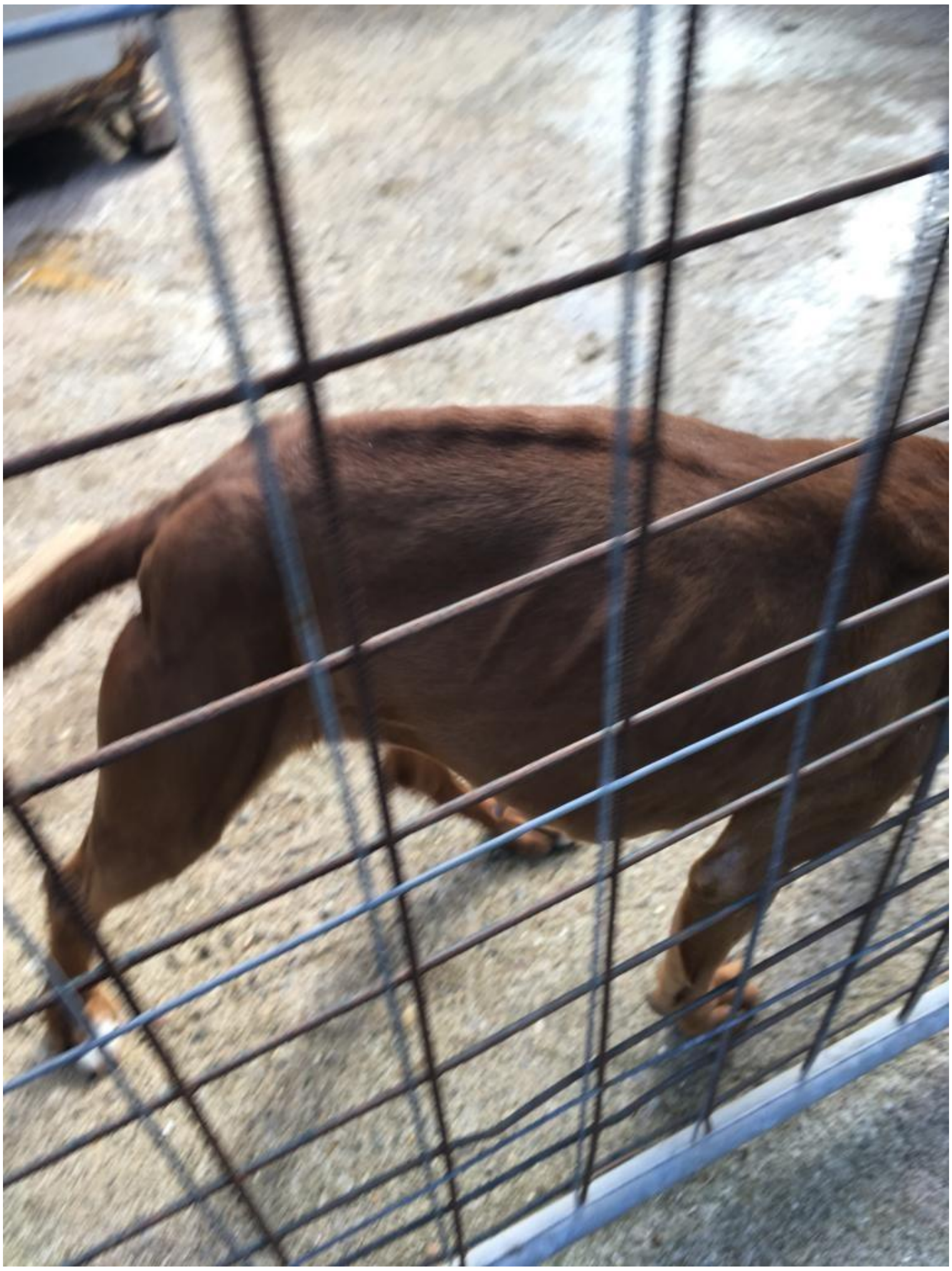

Figure S4. The dog in the photo represents a subject seized in February 2016. The animal shows spinal deviation, due to prolonged detention in small cages, and marked and widespread alopecia.

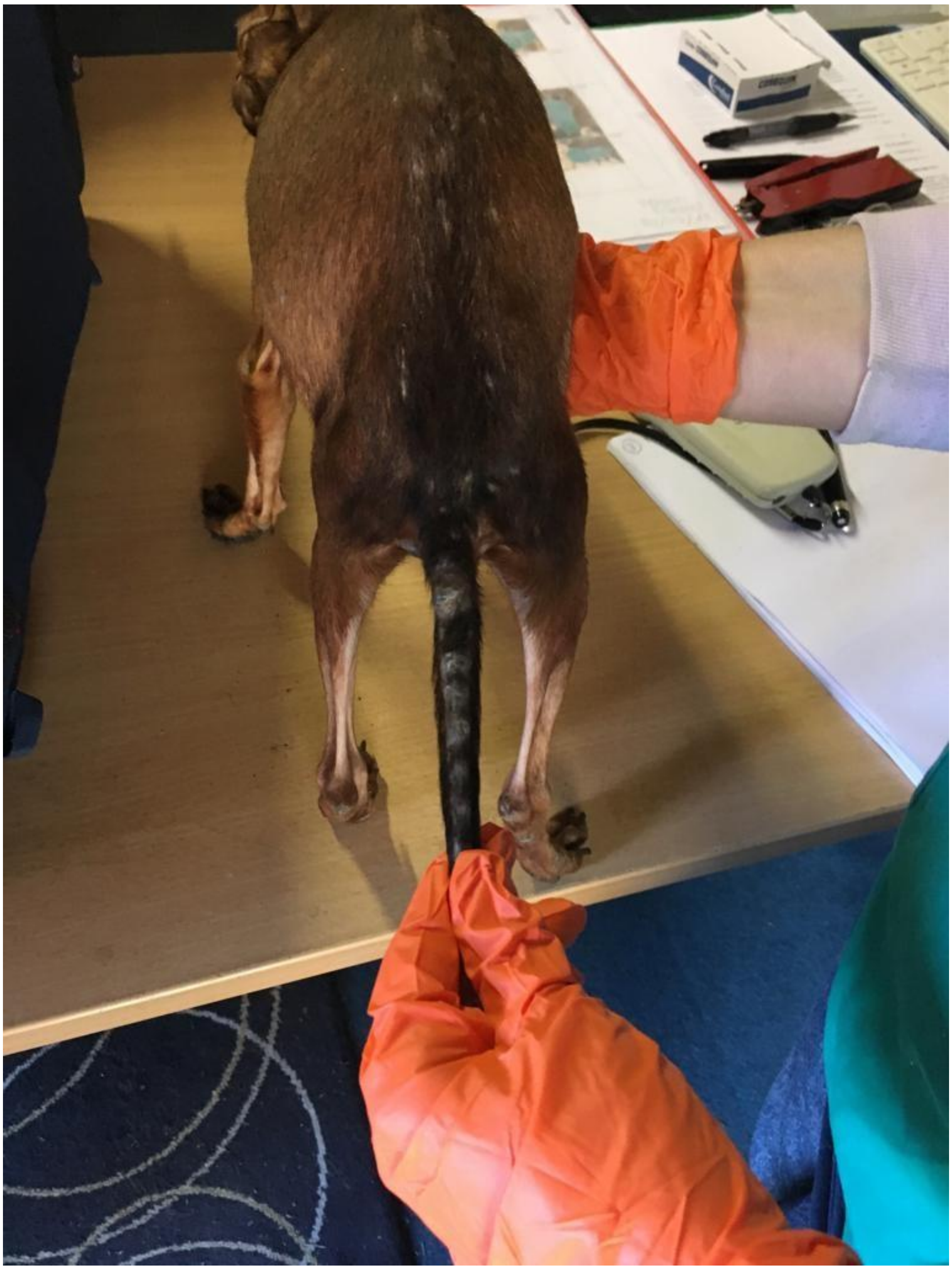

Figure S5. The dog in the photo represents a subject seized in February 2016 and shows deviation of the spine, due to the prolonged detention in small cages. There are also circumscribed alopecic areas.

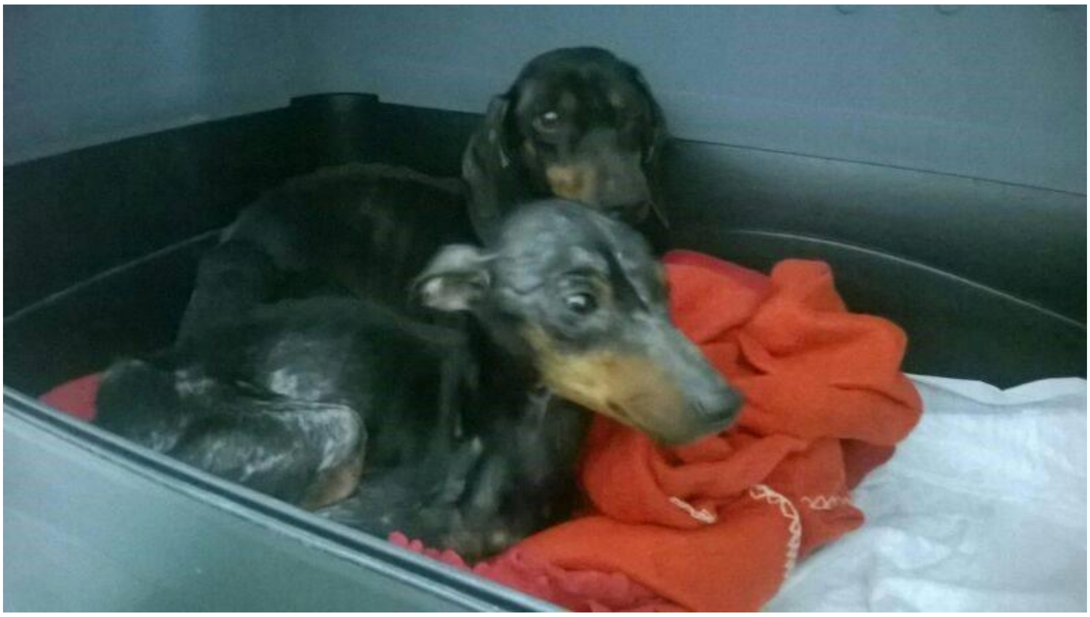

Figure S6. The photo shows two of the dogs seized on March 12, 2018. They show a widespread alopecia and a state of malnutrition and dehydration that in the foreground dog results in cachexia.

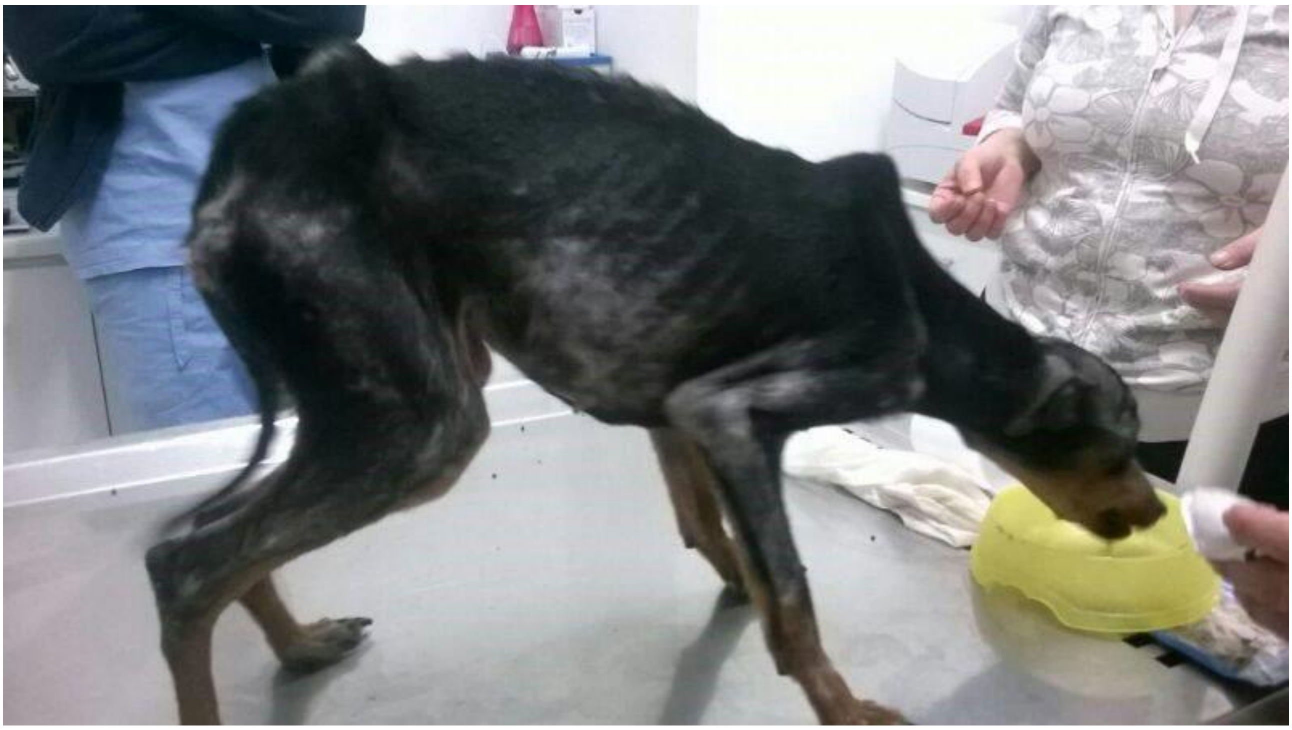

Figure S7. The dog in the photo, seized on March 12, 2018 shows cachexia, diffuse alopecia and marked muscle hypotrophy.

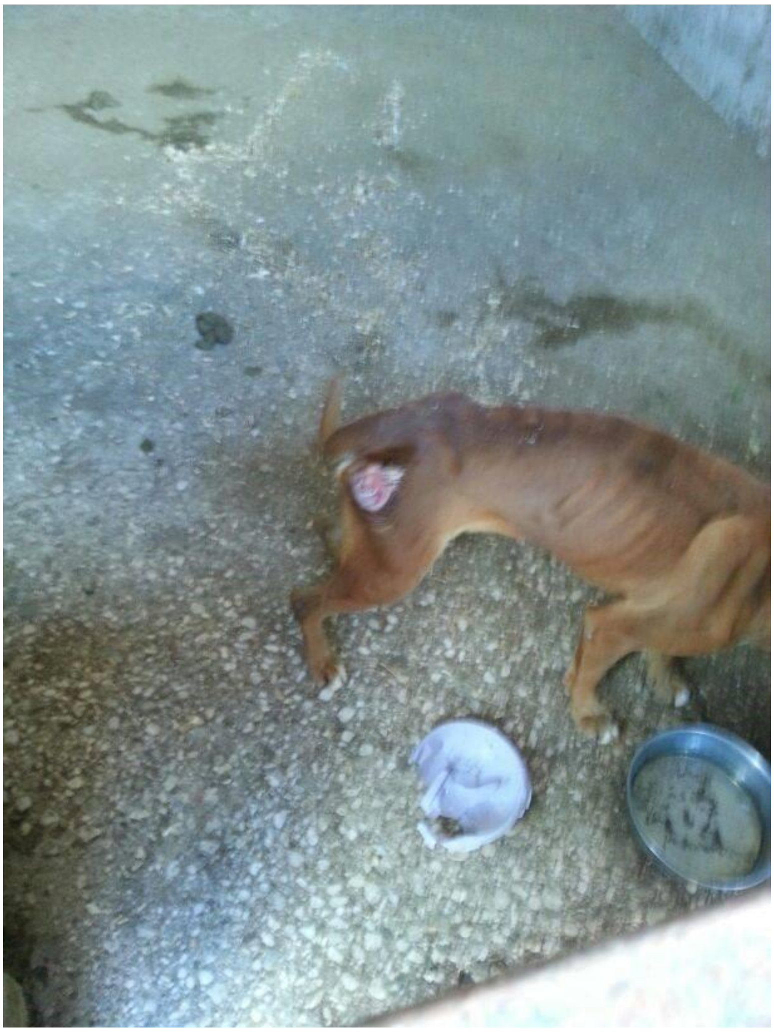

Figure S8. The dog in the photo, seized on March 12, 2018 shows signs of intraspecific bite injury.
